# Multitarget Approach to Cardiogenic Shock after Acute Myocardial Infarction: Extracorporeal Life Support (ECLS) and Beyond

**DOI:** 10.3390/membranes11020087

**Published:** 2021-01-27

**Authors:** Federico Pappalardo, Giulia Malara, Andrea Montisci

**Affiliations:** 1 Department of Anesthesia and Intensive Care, IRCCS ISMETT, UPMC Italy, 90127 Palermo, Italy; 2 Cardiothoracic Center, Department of Anesthesia and Intensive Care, Istituto Clinico Sant’Ambrogio, 20149 Milan, Italy; julia785@hotmail.it (G.M.); montisci.andrea@yahoo.it (A.M.)

**Keywords:** cardiogenic shock, acute myocardial infarction, extracorporeal life support

## Abstract

Cardiogenic shock following acute myocardial infarction is associated with high mortality, substantially unchanged for the previous 20 years. Several approaches have been sought to achieve a therapeutic breakthrough, from myocardial revascularization strategies to the use of mechanical circulatory support. Many issues are, as yet, unresolved. Systemic inflammation seems to play a key role but is still lacking in effective therapies, and is potentially compounded by the death spiral of hypoperfusion and/or artificial devices. In this review, a multitarget approach to cardiogenic shock following acute myocardial infarction is proposed.

## 1. Epidemiology of Acute Myocardial Infarction and Cardiogenic Shock (CS-AMI)

Acute myocardial infarction (AMI) is the ischemic necrosis of myocardial cells resulting from a period of prolonged ischemia. AMI is defined by the simultaneous presence of elevated cardiac necrosis biomarkers and at least one of the following:

- symptoms related to ischemia;
- changes on an electrocardiogram (ECG), such as ST segment changes or new left bundle branch block;
- development of pathological Q waves on ECG;
- new regional wall motion abnormalities at imaging;
- demonstration of a coronary thrombus on angiogram or during autopsy.

AMI is classified into five types according to the underlying pathophysiology: type 1 (coronary atherothrombosis or plaque rupture), type 2 (imbalance between myocardial oxygen supply and demand), type 3 (cardiac death accompanied by ischemic symptoms or suspected new ischemic changes on the ECG), type 4 (AMI associated with percutaneous coronary interventions, PCI) and type 5 (AMI associated with coronary artery bypass grafting) [1].

The burden of ischemic heart disease on public health is very high, with nearly 1.8 million deaths annually and increasing incidence [2].

Reperfusion strategies have, in recent decades, significantly improved prognoses after AMI, and the current mortality rate in Europe is between 4% and 12% for ST-segment elevation myocardial infarction (STEMI) in the acute hospital setting, and approximately 10% in the first year.

Cardiogenic shock after AMI (CS-AMI), however, complicates 5–10% of cases of AMI [3], with conflicting results about its incidence [4,5].

The CS-AMI mortality rate is very high. The AMIS registry analyzed 4900 patients experiencing CS from 1997 to 2017, reporting a decreasing incidence of in-hospital mortality over the last 20 years, with a 36.6% mortality rate in 2017 [6]. However, the 6–12 month mortality remained unchanged, reaching 50% over the past two decades [7].

## 2. Cardiogenic Shock after AMI: Definition and the Concept of “Spectrum of Shock”

CS-AMI can be defined as persistent hypotension (systolic blood pressure of ≤ 90 mm Hg for > 30 min or use of vasoactive drugs), end-organ hypoperfusion (altered mental status, cold/clammy skin, oliguria or arterial lactate ≥ 2.5 mmol/L) and reduced cardiac function, caused by an acute myocardial infarction [8].

Cardiogenic shock (CS) patients constitute a heterogeneous population, ranging from those who experience symptoms and signs of peripheral hypoperfusion, to critically ill patients with severe multiorgan dysfunction, to patients with cardiac arrest and ongoing need for resuscitation.

This heterogeneity was depicted in the recent classification of CS-AMI developed by the Society for Cardiovascular Angiography and Interventions (SCAI) [9].

This classification recognized the existence of a spectrum of CS, summarized into five stages of shock labeled A–E, starting from patients who are at risk of developing CS due to the characteristics of the primary myocardial insult to patients with cardiovascular collapse, who need mechanical ventilation and circulatory support (Table 1) [9].

These stages can be assigned during the initial phase of admission to the intensive care unit (ICU) and are meant to be based on standard clinical assessments. This classification, beyond its ease of application, reinforces the fact that CS-AMI must be considered as a dynamic clinical condition, in which the clinical deterioration has to be anticipated rather than addressed once it occurs. Higher SCAI classification has been recently associated with lower 30-day survival. A retrospective study applied this classification to stratify 1007 consecutive CS-AMI patients. The survival probability was 96.4% (95% CI 93.7–99.0%) in class A, 66.1% (95% CI 50.2–87.1%) in class B, 46.1% (95% CI 40.6–52.4%) in class C, 33.1% (95% CI 26.6–41.1%) in class D, and 22.6% (95% CI 17.1–30.0%) in class E [10].

## 3. The “Good Outcome” of Cardiogenic Shock after AMI

The outcomes of CS cannot be considered only in terms of survival from the acute event. Following the reduction of AMI mortality, the incidence of heart failure after AMI has been increasing over the last three decades [11]. The long-term follow-up of two RCT on CS-AMI provided additional insights. Indeed, a six-year follow up of the SHOCK trial showed a 28% increase in mortality compared with the 30-day results [12]. These data are consistent with the six-year follow up in the IABP-SHOCK II trial [13], and reinforce the evidence that the first 30-days act as a watershed. The functional status of CS survivors is good, as ≈90% of survivors in the IABP SHOCK and CULPRIT-SHOCK trials were in New York Heart Association class I or II at one-year follow up [12,14].

A significant percentage of patients after AMI, however, evolve towards adverse cardiac remodeling. A recent study showed that left ventricular remodeling (LVR) was observed in more than 30% of patients one year after AMI, and that LVR was strongly associated with hospitalization for HF [HR 2.52, (1.23–5.17)] and cardiovascular death [2.52 (1.45–4.36)] [15].

All these aspects have to be included in the therapeutic panel of CS, overcoming the simplistic target of short-term survival. This ambitious objective can be achieved through the deployment of a multifaceted approach that includes myocardial reperfusion, hemodynamics stabilization, reduction of mechanical circulatory support (MCS)-associated complications, ventricular unloading, and control of local and systemic inflammation to limit infarct size and prevent LVR [16,17].

## 4. Pathophysiology of AMI-Related Systemic Inflammation and Therapeutic Targets

The availability of an effective and timely therapy, i.e., myocardial revascularization, which is applied in the great majority of cases but which is not sufficient to save patients in cardiogenic shock, is a paradox of the stagnant mortality of CS-AMI [18].

Therefore, several directions have been sought to achieve a therapeutic breakthrough, including prompt recognition of the “at risk” and “early-stage” patient, implementation of protocols, proper timing and management of mechanical circulatory support application, multidevice approaches, and adjunctive therapies.

**Figure 1:**
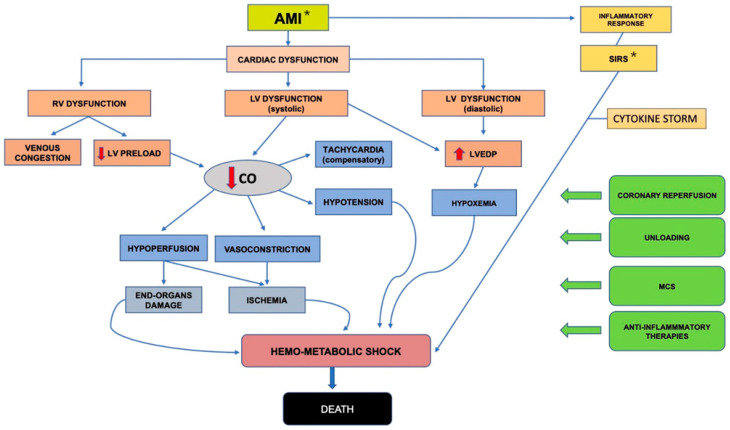
Pathophysiology of cardiogenic shock after acute myocardial infarction. *AMI: Acute Myocardial Infarction; RV: Right Ventricle; LV: Left Ventricle; LVEDP: Left Ventricular End-diastolic Pressure; *SIRS: Systemic Inflammatory Response Syndrome; CO: Cardiac Output; MCS: Mechanical Circulatory Support.

The pathophysiology of CS-AMI includes more than the reduction of cardiac output, involving multiple factors, i.e., primary myocardial damage, the systemic effects of the reduced cardiac output, and the local and systemic inflammatory response (Figure 1).

A landmark study [19] showed that in patients who died of cardiogenic shock after AMI, pathological examination showed extensive left ventricular necrosis, with more than 50% of the myocardium being involved. The extension of the infarcted area was shown to be secondary to the primary insult (acute ischemia triggered by coronary flow obstruction) and to the subsequent reduction of coronary flow mediated by reduction of cardiac output and coronary perfusion, causing ongoing ischemia. The reduced cardiac output induces myocardial and systemic hypoperfusion, causing the release of endogenous catecholamines and the production of pro-inflammatory cytokines [20].

Tissue-level ischemia is further worsened by the action of catecholamines, which induce arrhythmias and increase myocardial oxygen consumption.

Finally, CS moves from an initial hemodynamic phenomenon to “hemo-metabolic shock” [21], which is no longer responsive to the restoration of cardiac output and systemic flow.

Several recent studies have focused on the contribution of inflammation to AMI [22], highlighting the central role of a component of innate immune response, the inflammasome, a macromolecular protein complex that regulates the activation of caspase 1 and the production and secretion of pro-inflammatory cytokines such as IL-1β and IL-18 [23].

After the onset of coronary obstruction, myocardial ischemia progresses rapidly to necrosis of the cardiomyocytes. The direct damage secondary to acute ischemia and the paradoxical damage induced by reperfusion (ischemia-reperfusion injury) trigger a local and a systemic inflammatory response. According to Frangogiannis et al. [22], post-AMI inflammatory response is composed of three phases:

The alarm phase is characterized by release of damage-associated molecular patterns (DAMPs); DAMPs are interpreted by the innate immune system as danger signals, through the interaction with Pattern Recognition Receptor (PPR). The activation of PPRs by DAMPs ultimately results in a downstream signaling, leading to the production of several proinflammatory factors, chemokines, and cell adhesion molecules.

Secondly, cardiomyocyte necrosis triggers both a systemic response, mobilizing bone marrow-derived immune cells, and a local reaction, leading to recruitment of circulating inflammatory cells that serve to clear the infarct area from dead cells and matrix debris.

The humoral phase includes the production of cytokines. Interleukin-1 (IL-1), Tumor Necrosis Alpha (TNF-α), and IL-6 are the main promoters of inflammatory response in AMI. During AMI, the expression of cytokines, both in the ischemic and in the border zone, increases significantly [4]. On top of that, the activation of the complement system participates in the amplification of humoral and cell-mediated response.

Finally, the resolution phase is associated with the suppression of pro-inflammatory signaling and clearance of the leukocyte infiltrate.

Prolonged and exaggerated inflammatory pathways and ischemia-reperfusion injury, however, can cause additional damage and favor chronic adverse remodeling [24].

On clinical grounds, emerging evidence indicates that the level of systemic inflammation in the acute phase of AMI impacts on the prognosis. Several substudies of an IABP SHOCK trial showed that the levels of multiple cytokines, such as INF-γ, tumor necrosis factor-α (TNF-α), macrophage inflammatory protein-1β (MIP-1β), granulocyte-colony stimulating factor (G-CSF), and monocyte chemoattractant protein-1β (MCP-1β) have a prognostic role, with higher levels associated with higher mortality risk [25].

A recent study showed that the levels of IL-6, IL-10 and MCP-1, but not IL-1b, were associated with shock severity in a heterogenous cohort of CS patients [26], and previous studies reported that inflammation-associated cytokines as TNF-α, IL-6 and IL-1Ra are significantly elevated in patients with CS-AMI compared to patients with uncomplicated MI [27]. These data strongly support research on inflammation control during the acute phase of AMI.

The role of IL-1 is of particular interest. IL-1 family is upregulated in AMI, leading to ventricular dysfunction and inflammation. IL-1α and IL-1β are both agonists of the IL1R; IL-1α is released by necrotic cardiomyocytes and functions as an alarm signal, triggering a postinfarction inflammatory reaction, while IL-1β is produced by the leukocytes invading the infarct area after myocardial infarction [28]. Two IL-1 pathway antagonists have been widely studied: anakinra, that acts as a competitive inhibitor of IL-1α/ IL-1β, and canakinumab, an IL-1β antibody, blocking only the IL-1β pathway.

In previous studies [29,30], the administration of anakinra was associated with a significant reduction of inflammation markers, and the recent RCT VCUART3 showed that the reduction in inflammatory signaling was accompanied by significant reductions in new-onset heart failure and hospitalization for heart failure.

Interesting results also emerged from a large RCT, the Canakinumab Anti-inflammatory Thrombosis Outcomes Study (CANTOS) [31]. In this study 10,061 patients, with prior AMI and evidence of systemic inflammation, as determined by elevated serum CRP, were randomized to receive either placebo or a monoclonal antibody against IL-1β, canakinumab, in combination with standard care.

At 48 months, patients treated with canakinumab exhibited a reduction in recurrent cardiovascular events, and this benefit was closely related to the suppression of inflammation, as shown by a reduction in CRP. In the canakinumab group, an increased incidence of fatal infections and sepsis was observed.

A recent study showed the association between the levels of circulating IL-1b and all-cause mortality in patients with STEMI. The authors reported that IL-1b measured at admission was independently associated with the risk of mortality and recurrent major adverse cardiac events (MACEs) at 90 days, and that the higher tertile of IL-1b concentration was associated with the higher mortality at 90 days [32].

Colchicine represents an accessible, relatively safe, and well tolerated drug that deeply influences cellular function. Colchicine has multiple mechanisms of actions, affecting the assembly of inflammasome and the release of many proinflammatory cytokines, as IL-1 and IL-6 [33]. The potential benefits of colchicine in stable coronary artery disease and AMI were highlighted in previous studies [34,35].

Recently, two RCTs have been published. The COLCOT trial [36] enrolled 4745 patients, of which 2366 were assigned to the colchicine group (0.5 mg once daily) and 2379 to the placebo group. The median follow-up was 22.6 months. The primary composite endpoint of death from cardiovascular causes, resuscitated cardiac arrest, AMI, stroke, or need for urgent revascularization occurred in 5.5% of the patients in the colchicine group, as compared with 7.1% of those in the placebo group (hazard ratio, 0.77; 95% confidence interval [CI], 0.61 to 0.96; *p* = 0.02). The incidence of diarrhea was not different between groups, whereas pneumonia was reported as a serious adverse event in 0.9% of the patients in the colchicine group and in 0.4% of those in the placebo group (*p* = 0.03).

The findings of the COLCOT trial appear to be in conflict with the Australian COPS trial, a multicenter, randomized, double-blind, placebo-controlled trial [37] that enrolled 795 patients adult patients with acute coronary syndrome (ACS). In that study, 396 patients received colchicine (0.5 mg twice daily for the first month, then 0.5 mg daily for 11 months) and 399 received a placebo. The minimum follow-up was 12 months. The primary outcome was a composite of all-cause mortality, ACS, unplanned, ischemia-driven urgent revascularization, and noncardioembolic ischemic stroke. No differences in the primary endpoint were observed between groups; looking at the individual components, there was a significant increase of total mortality (eight versus one, *p* = 0.023) and noncardiovascular death in the colchicine group (five versus zero, *p* = 0.024). The rate of reported adverse effects was not different, and such symptoms were predominantly gastrointestinal.

Particular attention should be paid to the differences between the two studies. The sample size of the COLCOT study was much larger; the inclusion criteria, the dose and schedule of colchicine administration were different; the positive composite outcome of the COLCOT study was mostly driven by a reduction in stroke and urgent repeated revascularization. Of note, all these trials enrolled stable patients, without signs of shock.

Despite not univocal results coming from RCTs, the importance to target inflammation has repeatedly demonstrated. The link between systemic inflammatory response, SCAI stage and in-hospital and one1-year mortality was recently highlighted [38]. Notably, 8999 patients admitted to a cardiac ICU with CS secondary to various etiologies were retrospectively analyzed. The outcome of the study was to evaluate the association of SIRS with in-hospital and one-year mortality across the different SCAI stages. SIRS was present in 33.9% of patients, with an increasing prevalence in the more advanced SCAI stages. The presence of SIRS conferred an increase of both in-hospital and 30-day mortality for any given SCAI stage, except for stage E.

## 5. Extracorporeal Life Support

In CS-patients, the application of mechanical circulatory support (MCS) systems unavoidably provides additional triggers for dysregulated inflammatory response. Indeed, the interaction between blood and biomaterials triggers contact activation of coagulation through the activation of factor XIII, which, in turn, activates the bradikinine-kallikreine system that enhances the intrinsic coagulation pathway, leading to uncontrolled thrombin generation. In parallel, increased hydrolysis of complement molecule C3 leads to the production of C3a and C5a, forming the membrane attack complex (MAC), which is able to induce a transmembrane pore leading to cell lysis [39].

These systems, once activated, promote the production of pro-inflammatory cytokines and elicit the activity of leukocytes, platelets and the vascular endothelium. A comprehensive review of ECLS-induced inflammation is given in [39].

Experimental and clinical evidence that inflammation in coronary artery disease plays an important role has been presented multiple trials [36,40] with encouraging results; however, none of these trials included CS patients, and the organization of randomized clinical trials in CS-AMI is afflicted by well-known limitations and obstacles [41].

Extracorporeal remotion of inflammatory mediators is a promising and safe technique to treat systemic inflammation in patients on ECLS and suffering from shock, and its application has a strong rationale as an adjunctive therapy [42,43]. The number of studies on these topics is increasing, but such studies still suffer from limited sample sizes, single center designs and high heterogeneity.

## 6. Conclusions

CS is a pleiotropic disease, in which multiple, tightly linked events combine, leading to negative outcome. The first step to achieving a therapeutic breakthrough is to realize that no single therapy has been proven to be effective and none is free of risk of inducing additional damage.

CS patients show significant interindividual differences, that, at present, we cannot adequately capture, either in the clinical practice or in clinical studies.

Recent research has opened multiple promising fronts for the control of inflammation and unloading-promoted recovery. Particular attention also must be given to the conflict arising from the ECLS application and its detrimental effects on systemic inflammation and associated complications.

MCS ensuring forward flow and unloading can be less rapid or effective in determining systemic reperfusion, but the potential benefit of slower restoration of blood flow on ischemia-reperfusion injury should be explored. Therefore, a multidevice strategy with the concomitant use of different MCS systems, such as Impella and ECMO (ECPELLA or ECMELLA), appears particularly appealing, and several studies are giving clinical strength to this approach [44,45].

There are strong expectations around RCT focused on MCS in CS-AMI, i.e., EURO-SHOCK [46], ECLS-SHOCK [47] and DANGER Shock [48], ANCHOR [49], but the lessons learned from previous RCT should prepare us for a possible null result. In this sense, a global rethinking about the sources of evidence for CS therapy may be necessary.

## Figures and Tables

**Table 1:** Society of Cardiovascular Intervention classification of cardiogenic shock after Acute Myocardial Infarction.

Stage	Definition	Physical Exam/Bedside Findings	Biochemical Markers	Hemodynamics
**A** At risk	A patient who is not currently experiencing signs or symptoms of CS but is at risk for its development. These patients may include those with large acute myocardial infarction or prior infarction acute and/or acute on chronic heart failure symptoms.	Normal Jugular Venous Pressure Lung sounds clear Warm and well perfused • Strong distal pulses • Normal mentation	Normal labs • Normal renal function • Normal lactic acid	Normotensive (SBP ≥ 100 or normal for pt.) If hemodynamics done • cardiac index ≥ 2.5 • CVP < 10 • PA sat ≥ 65%
**B** Beginning CS	A patient who has clinical evidence of relative hypotension or tachycardia without hypoperfusion.	Elevated JVP Rales in lung fields Warm and well perfused • Strong distal pulses • Normal mentation	Normal lactate Minimal renal function impairment Elevated BNP	SBP < 90 OR MAP < 60 OR > 30 mmHg drop from baseline Pulse ≥ 100 If hemodynamics done • cardiac index ≥ 2.2 • PA sat ≥ 65%
**C** Classic CS	A patient that manifests with hypoperfusion that requires intervention (inotrope, pressor or mechanical support, including ECMO) beyond volume resuscitation to restore perfusion. These patients typically present with relative hypotension.	May Include Any of: Looks unwell Panicked Ashen, mottled, dusky Volume overload Extensive rales Killip class 3 or 4 BiPap or mechanical ventilation Cold, clammy Acute alteration in mental status Urine output < 30 mL/h	May Include Any of: Lactate ≥ 2 Creatinine doubling OR > 50% drop in GFR Increased LFTs Elevated BNP	May Include Any of: SBP < 90 OR MAP < 60 OR > 30 mmHg drop from baseline ANDdrugs/device used to maintain BP above these targets Hemodynamics • cardiac index < 2.2 • PCWP > 15 • RAP/PCWP ≥ 0.8 • PAPI < 1.85 • cardiac power output ≤ 0.6
**D** Deteriorating/doom	A patient that is similar to category C but are getting worse. They have failure to respond to initial interventions.	Any of stage C	Any of Stage C AND: Deteriorating	Any of Stage C AND: Requiring multiple pressors ORaddition of mechanical circulatory support devices to maintain perfusion
**E** Extremis	A patient that is experiencing cardiac arrest with ongoing CPR and/or ECMO, being supported by multiple interventions.	Near Pulselessness Cardiac collapse Mechanical ventilation Defibrillator used	“Trying to die” CPR (A-modifier) pH ≤ 7.2 Lactate ≥ 5	No SBP without resuscitation PEA or refractory VT/VF Hypotension despite maximal support
